# Exploring the role of Paraoxonase-2 in bladder cancer: analyses performed on tissue samples, urines and cell culturess

**DOI:** 10.18632/oncotarget.15674

**Published:** 2017-02-24

**Authors:** Tiziana Bacchetti, Davide Sartini, Valentina Pozzi, Tiziana Cacciamani, Gianna Ferretti, Monica Emanuelli

**Affiliations:** ^1^ Department of Life and Environmental Sciences, Polytechnic University of Marche, Ancona, Italy; ^2^ Department of Clinical Sciences Polytechnic University of Marche, Ancona, Italy; ^3^ New York-Marche Structural Biology Center (NY-MaSBiC), Polytechnic University of Marche, Ancona, Italy

**Keywords:** paraoxonase-2, oxidative stress, bladder cancer, biomarker, antioxidant

## Abstract

The enzyme paraoxonase-2 (PON2) is ubiquitously expressed and exerts its antiapoptotic and antioxidative functions in several intracellular compartments.

The aim of this study is to investigate the role of PON2 in bladder cancer (BC). The expression levels of PON2 in paired tumor and normal bladder tissue samples and in urinary exfoliated cells from patients affected with BC and healthy donors were evaluated. Moreover, the effect of PON2 overexpression on tumor cell proliferation and susceptibility to oxidative stress was investigated in human bladder cancer cell line T24.

Our results showed that PON2 expression levels were significantly higher in BC compared with non-tumor tissue. In urinary exfoliated cells from BC patients, PON2 mRNA levels showed an inverse correlation with tumor stage (pT). Moreover, PON2 overexpression in T24 cells led to a significant increase in tumor cell proliferation and resistance to oxidative stress.

The results obtained showed that PON2 could represent a molecular biomarker for bladder cancer and suggest a potential role of the enzyme as a prognostic factor for this neoplasm.

## INTRODUCTION

Paraoxonase-2 (PON2), a member of the multigene family of paraoxonases (PONs), is expressed in various tissues and cells. PON2 exerts its functions in intracellular environment [1–3]. In vascular cells, PON2 is localized in mitochondria, in endoplasmatic reticulum (ER) and in nuclear lamina. PON2 exerts a protective role against overwhelming levels of reactive oxygen species (ROS) production within the mitochondrial respiratory chain [2]. Thereby, release of cytochrome c and caspase activation is minimized, which finally counteracts induction of mitochondria-induced apoptosis [4–6]. The antiapoptotic and antioxidant functions of PON2 in cardiovascular and neurodegenerative diseases have been subject of intensive research [3, 7–11]. Only few studies have been focused on its biological function in cancer cells [2, 6, 12]. Till now, upregulated levels of PON2 have been detected in different types of cancer cells, such as hepatocellular carcinoma, prostate cancer and pediatric acute lymphoblastic leukemia, and it has been suggested a possible involvement of PON2 in apoptotic escape of tumor cells [6, 13–15]. PON2 knockdown in the tumor cell lines K562 (leukemia) and A549 (lung cancer) initiated apoptosis [6], suggesting that PON2 may act as a target for cancer therapy in certain malignancies and fulfill an outstanding function for tumor cell survival.

Bladder cancer (BC) is the 7th most common cancer in men and the 17th most common in women, worldwide. BC displays the highest recurrence rate of any other solid tumor; indeed, the most of non-muscle invasive forms relapses or progresses to a muscle invasive disease. Since muscle invasive BC is often associated with the presence of metastases, this tumor form contributes to the vast majority of cancer-specific deaths. Therefore, there is an urgent need to identify biomarkers for BC, which can be used to perform an early diagnosis of this cancer as well as to select high-risk patients.

The aim of this study is to investigate the physio-pathological role of PON2 in bladder cancer. Therefore, we evaluated the expression levels of PON2 in paired tumor and normal bladder tissue samples from patients affected with BC, most of which underwent radical cystectomy for treatment of advanced disease (pT3-4). Moreover, PON2 expression was investigated in urinary exfoliated cells obtained from a large cohort of BC patients, mainly with early stage neoplasms (pTa-1), and healthy donors, in order to explore the diagnostic/prognostic power of PON2 expression levels determination for early and non-invasive diagnosis of bladder cancer and for the prediction of the clinical outcome. Finally, the effect of PON2 overexpression on tumor cell proliferation and susceptibility to oxidative stress was investigated in human bladder cancer cell line T24.

## RESULTS

### PON2 expression in tissue samples

**Figure 1:**
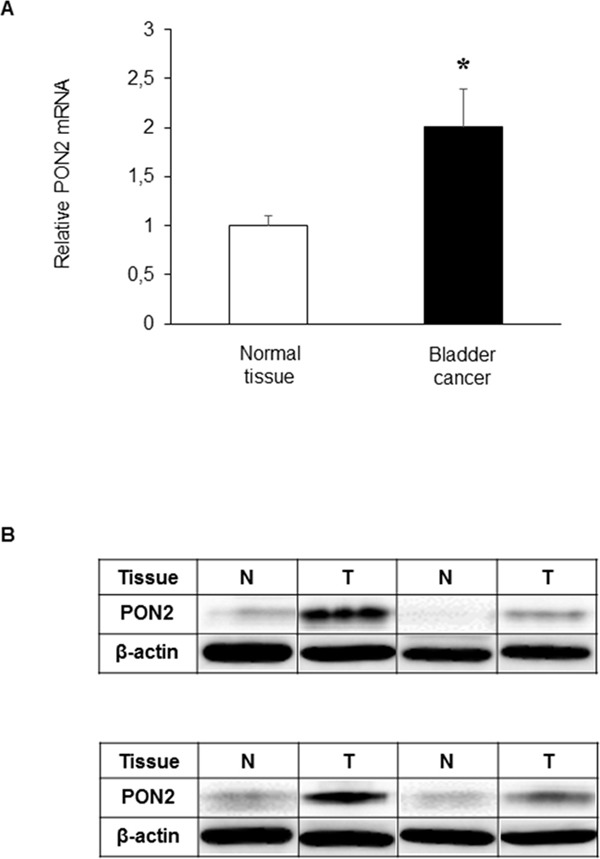
PON2 expression in bladder tissue PON2 mRNA and protein levels were determined in paired normal (N) and tumor (T) tissue samples from patients with BC, by Real-Time PCR **(A)** and Western blot analysis **(B)**, as described under Materials and Methods. Values are reported as mean ± standard deviation (*p<0.05).

Results obtained from Real-Time PCR analyses performed on tissue samples showed that PON2 expression levels were significantly (p<0.05) higher (2.01-fold) in BC compared with those detected in normal looking tissue (Figure 1A). To confirm these results, PON2 expression was detected at protein level by Western blot analysis in a representative cohort of samples. Consistent with the results of Real-Time PCR, lanes loaded with equal protein amounts showed markedly increased PON2 expression in tumor samples compared to that in matched normal tissues, which in some specimens showed a faintly detectable band (Figure 1B). Statistical analyses demonstrated that there was no significant correlation between PON2 expression level in BC and gender (p=0.294), age (p=0.161), pT (p=0.328) and lymph node metastasis (p=0.536) (Supplementary Data 1).

### PON2 expression in urine samples

**Figure 2:**
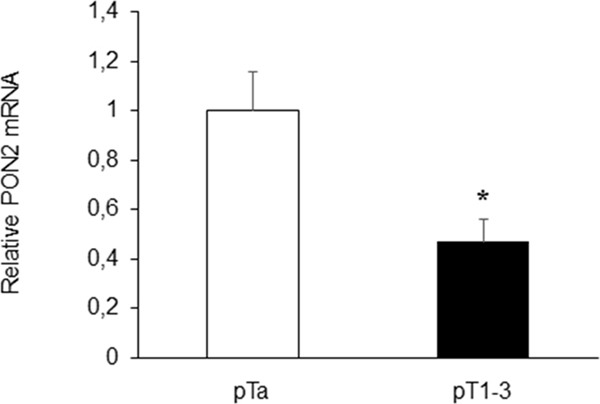
Correlation between urinary PON2 expression level and pT Statistically significant inverse correlation was found between PON2 mRNA expression in urine samples of BC patients and pT. Values are reported as mean ± standard deviation (*p<0.05).

Analyses performed on urine specimens revealed that PON2 expression did not differ significantly (p=0.917) between pathological samples and healthy controls. Moreover, in patients affected with BC, urinary PON2 expression levels did not correlate with gender (p=0.783), age (p=0.644) and histological grading (p=0.488) (Table 1). Interestingly, the parameter pT, which describes the size and extent of primary tumor, showed an inverse correlation with PON2 mRNA levels. In particular, PON2 expression was significantly (p<0.05) higher (1.00 ± 0.16) in patients with disease confined within the bladder basement membrane (pTa) compared with those (0.47 ± 0.09) affected with tumors invading subepithelial connective tissue or extending outside bladder (pT1-3) (Figure 2).

**Table 1:** Linkage between urinary PON2 expression and clinicopathologic parameters of BC patients

Parameter	Category	PON2 level ^a^	p value ^b^
Gender	Male	1.00 ± 0.20	0.783
	Female	0.83 ± 0.10	
Age (years)	<75	1.00 ± 0.17	0.644
	≥75	0.78 ± 0.16	
T classification	pTa	1.00 ± 0.16	<0.05
	pT1-3	0.47 ± 0.09	
Histological grading	PUNLMP-low grade ^c^	1.00 ± 0.17	0.448
	High grade	0.76 ± 0.15	

^a^ Values represent mean ± standard deviation.

^b^ Mann-Whitney U test was used for comparison of two groups.

^c^ PUNLMP = papillary urothelial neoplasm of low malignant potential.

### Efficiency of PON2 overexpression in T24 cells

**Figure 3:**
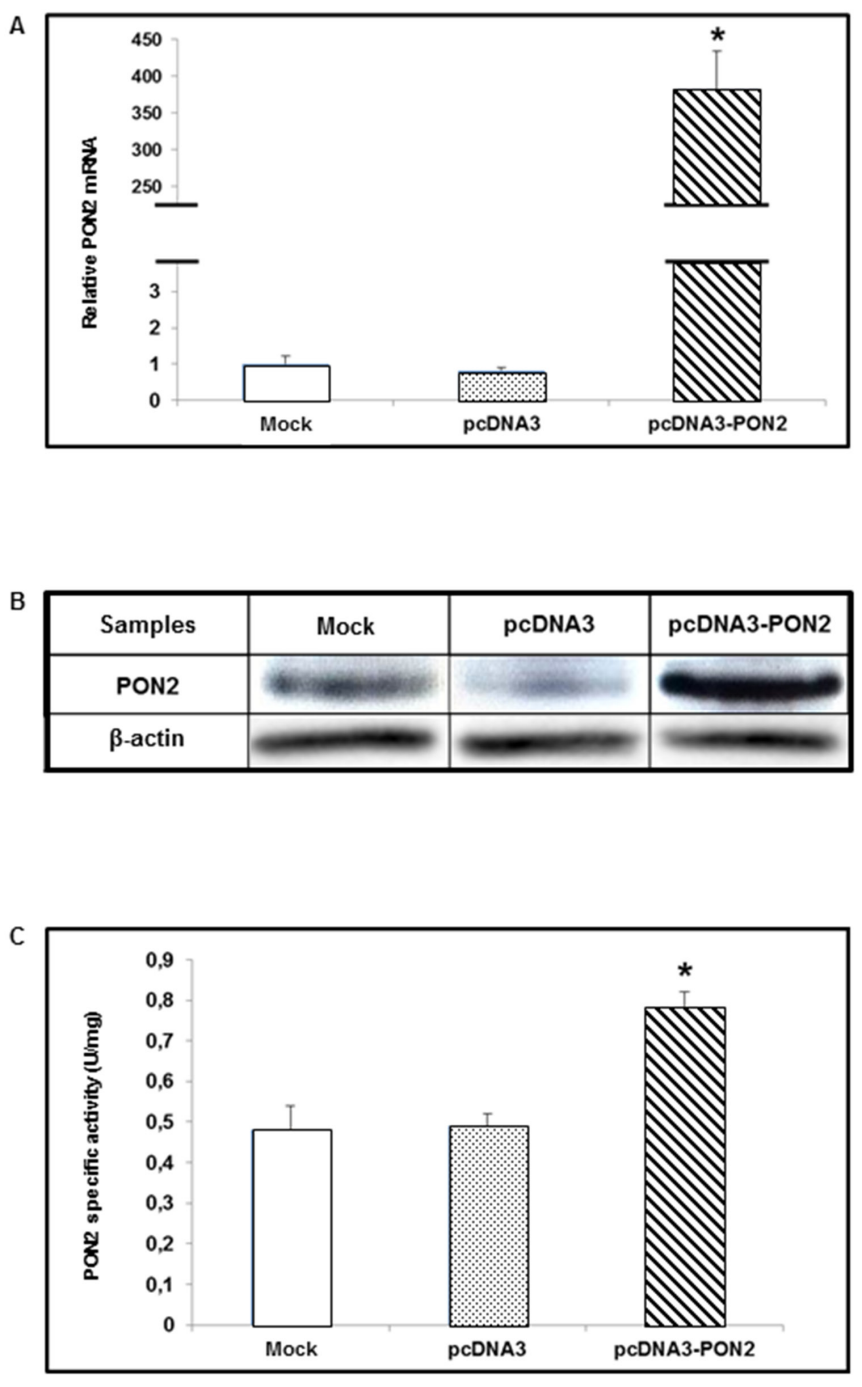
PON2 expression levels in T24 cells T24 cells were transfected with pcDNA3-PON2, with pcDNA3, or treated with transfection reagent only (mock). PON2 mRNA levels were evaluated by Real-Time PCR in transfected (pcDNA3 and pcDNA3-PON2) compared with mock cells **(panel A)**. Lysates, obtained from transfected and mock cells, were analyzed by Western blot to measure PON2 protein expression levels **(panel B)**. PON2 lactonase activity was determined using dihydrocoumarin (DHC) as substrate **(panel C)**. All values reported in panels A and C are expressed as mean ± standard deviation (*p<0.05).

In order to modulate PON2 expression for functional assays, T24 cell line was transiently transfected with the PON2 expression vector (pcDNA3-PON2), and control cells were transfected with the empty vector (pcDNA3) or treated with transfection reagent only (mock), as described in Materials and Methods. Forty-eight hours after transfection cells were harvested. To evaluate enzyme overexpression, PON2 mRNA, protein and catalytic activity levels were analyzed by Real-Time PCR, Western blot and enzyme assay, respectively. Compared with mock and pcDNA3-treated cells, T24 transfected with pcDNA3-PON2 displayed significantly increased PON2 expression levels. Real-Time PCR showed a significant (p<0.05) upregulation of PON2 in cells transfected with pcDNA3-PON2 plasmid (381.52 ± 46.78) compared with pcDNA3-treated (0.82 ± 0.07) and mock (1.00 ± 0.21) cells (Figure 3A). PON2 overexpression was confirmed at protein level by Western blot analysis. Lanes loaded with equal protein amounts displayed markedly increased PON2 expression in cells treated with pcDNA3-PON2 (Figure 3B). In keeping with results obtained by Real-Time PCR and Western blot analysis, PON2 specific activity levels, expressed in U/mg protein, were significantly (p<0.05) higher in cells transfected with the pcDNA3-PON2 (0.78 ± 0.04) compared with pcDNA3 (0.49 ± 0.03) and mock (0.48 ± 0.06) (Figure 3C).

### Effect of PON2 overexpression on proliferation of T24 cells and susceptibility to oxidative stress

**Figure 4:**
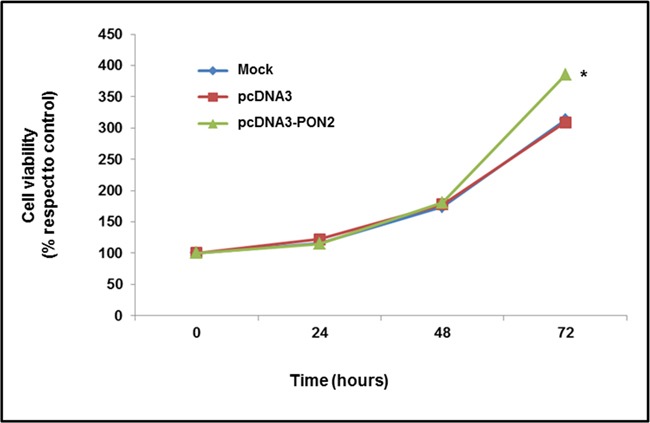
Evaluation of T24 cell proliferation *In vitro* effect of PON2 overexpression on cell proliferation was assessed by MTT assay. Cell growth was evaluated in mock and transfected cells after 24, 48 and 72 h of incubation. All values are expressed as mean ± standard deviation (*p<0.05).

To examine the role of PON2 in tumor cell metabolism, and analyze the biological effect associated with enzyme upregulation, pcDNA3-PON2 vector was introduced into T24 cells, and cell viability was then assayed. The effect of PON2 overexpression on cell proliferation was evaluated by MTT assay. As shown in Figure 4, enzyme upregulation led to a significant (p<0.05) increase in cell growth of T24 cells at 72h time point. To confirm at molecular level this phenotypic trait, the expression of the cell proliferation marker MIB-1 (Ki-67) was evaluated by Real-Time PCR. Results obtained showed a significant upregulation (p<0.05) of MIB-1 in PON2 overexpressing cells (3.92 ± 0.13) compared with controls (1.00 ± 0.10).

**Figure 5:**
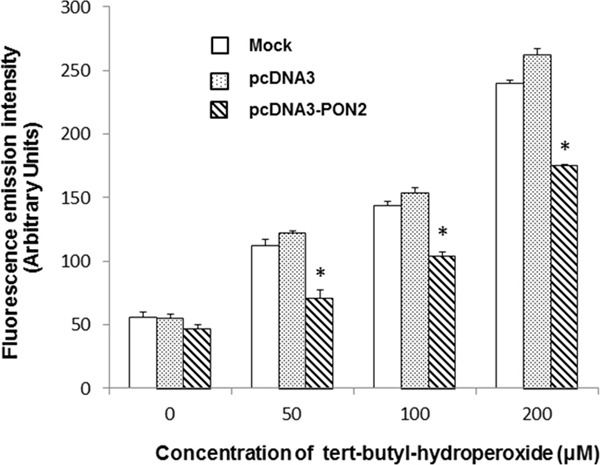
Intracellular ROS levels in T24 cells *In vitro* effect of PON2 overexpression on basal intracellular ROS levels and after treatment with different concentrations of tert-butyl hydroperoxide (TBHP) for 3 hours. All values are expressed as mean ± standard deviation (*p<0.05).

To assess the functional consequences of PON2 overexpression on ROS production, intracellular ROS levels were evaluated before and after incubation of T24 cells with the oxidant tert-butyl-hydroperoxide (TBHP). As shown in Figure 5, no significant difference was observed in basal ROS levels between cells overexpressing PON2 and controls. Conversely, upon treatment with different concentrations of TBPH, intracellular ROS production was significantly (p<0.05) lower in PON2 overexpressing cells compared with control cells (Figure 5).

## DISCUSSION

Our results demonstrated that PON2 expression levels were significantly higher in BC compared to those detected in adjacent normal looking tissue. The higher expression of PON2 in BC tissues is in agreement with previous studies that reported upregulation of PON2 in few types of human cancers [6, 13–15]. In this study, we also investigated for the first time PON2 expression in urine specimens from subjects affected by BC and healthy controls. Interestingly, PON2 mRNA levels showed an inverse correlation with the clinical parameter pT, which takes into account the size and the extent of primary tumor, thus suggesting a potential role for the enzyme in the early stages of the tumor.

To better investigate the role of PON2 in BC, enzyme overexpression has been induced in human urinary bladder cancer cell line T24. Results demonstrated that PON2 overexpression significantly increased T24 cell proliferation, highlighting that this gene might play an important role among the events promoting bladder tumorigenesis. Interestingly, MIB-1 levels were significantly higher in T24 cells overexpressing PON2 compared with those detected in control cells. Ki-67 is known to be present in the nuclei of cells in the G1, S, G2 phases as well in mitosis, while is not expressed in quiescent or resting cells (G0 phase). From this point of view, its upregulation in PON2 overexpressing T24 cells could represent one of the factors responsible for the detected high proliferative capacity [16].

Moreover, increased PON2 expression significantly counteracted the increase in cellular ROS production in response to oxidative stress triggered by TBHP. These data are in agreement with previous reports showing that PON2 overexpression is associated with reduced cellular ROS levels. Several studies demonstrated that PON2 protected macrophages, vascular and other cells against oxidative stress, whereas its downregulation reversed this effect [2, 3, 17]. Furthermore, animal studies have shown that mice subjected to adenovirus-mediated expression of PON2 (AdPON2) display an increased antioxidant capacity with lower levels of lipid hydroperoxides when compared to mice treated with either PBS or empty vector [18].

Several hypotheses could be advanced to explain the lower ROS production induced by oxidative stress triggered by TBHP in PON2 overexpressing cells. PON2 is a transmembrane protein and it has been reported that could directly interfere with lipid peroxidation products of cellular membrane lipids [19]. In fact, it has been reported that oxidized metabolites of polyunsaturated fatty acids could be physiological substrates of PONs [20]. Moreover, given the localization of PON2 in mitochondria, it has been demonstrated that PON2 is able to improve mitochondrial efficiency leading to reduced ROS production. PON2 has been shown to bind to Coenzyme Q10 that associates with Complex III in mitochondria, and PON2 deficiency causes mitochondrial dysfunction [5].

There is accumulating evidence that cancer cell response to apoptotic insults is regulated by the cellular redox status and it is established that oxidative stress is closely linked to cell death and cancer. Therefore, our results support the hypothesis that PON2 upregulation observed in BC tissues could represent an adaptive mechanism, which could enable the cancer cells to escape cell death and apoptosis.

In conclusion, although further studies are necessary to clarify the role of PON2 in BC cancer cell, our results suggest that the overexpression of this enzyme has an impact on BC cell proliferation and resistance to oxidative stress. In this light, PON2 silencing in BC cells may therefore represent a novel molecular approach to control tumor growth and its susceptibility to chemotherapeutics. The efficacy of this strategy is supported by results reported by previous studies demonstrating that PON2 knockdown induced apoptosis of several human tumor cell lines and enzyme overexpression increased chemotherapeutic resistance [6].

To our knowledge, this report is the first to evaluate PON2 expression in both bladder tissues and exfoliated urinary cells. Our results, although obtained from analyses performed on a limited cohort of patients, indicate that PON2 may represent a potential molecular biomarker for bladder cancer. Moreover, the identification of an inverse correlation between PON2 expression levels and pT suggests a potential role of this enzyme as a prognostic factor for BC.

## MATERIALS AND METHODS

### Patients and sample collection

A total of 17 patients, who underwent surgical treatment for BC at the Department of Clinical Sciences between January 2006 and October 2008, were analysed in this study. All samples were obtained at radical cystectomy. Pathological stage was assigned to the tumors using the 2010 TNM classification system. Grade was assigned according to 2004 WHO classification. Fresh tumor and adjacent normal looking tissues were collected at surgery, snap frozen in liquid nitrogen and stored at −80°C until use.

Spontaneously voided urine samples were collected from 41 patients with BC, before endoscopic treatment with transurethral resection (between January 2012 and July 2014) at the Department of Clinical Sciences, and from 55 age- and gender-matched healthy volunteers. Urine samples (50-100 ml) were centrifuged at 1200 xg for 15 min at 4°C to collect exfoliated cells. The urine cell pellet was snap frozen in liquid nitrogen and stored at −80°C until use.

Tables 2A and 2B list the characteristics of the patients involved in the studies carried out on tissue and urine samples, respectively. The study was performed in accordance with the Declaration of Helsinki and informed consent was obtained from all participants.

**Table 2A:** BC patients and clinicopathologic findings

Cases	17
Age (years), range (years)	69, 47-80
Gender (male:female)	15:2
T classification	
pT1	1
pT2	4
pT3	9
pT4	3
Lymph nodes	
N0	10
N+	7
Histological grading (2004 WHO classification)	
PUNLMP ^a^	0
Low grade	0
High grade	17

Analysis performed on tissue samples.

^a^ PUNLMP = papillary urothelial neoplasm of low malignant potential.

**Table 2B:** BC patients and clinicopathologic findings

Cases	41
Age (years), range (years)	75, 59-90
Gender (male:female)	37:4
T classification	
pTa	17
pT1	17
pT2	5
pT3	2
Lymph nodes	
N0	40
N+	1
Histological grading (2004 WHO classification)	
PUNLMP ^a^	1
Low grade	11
High grade	29

Analysis performed on urine samples.

^a^ PUNLMP = papillary urothelial neoplasm of low malignant potential.

### RNA extraction and cDNA synthesis

An aliquot of the frozen tissue (20–40 mg) was homogenized in a lysis buffer. Total RNA was extracted through the SV total RNA Isolation System (Promega, Madison, WI, USA). Total RNA was isolated from exfoliated urinary cells using the RNeasy Micro Kit (Qiagen, Hilden, Germany), according to the manufacturer's instructions.

Total RNA, obtained from both tissue and urine samples, was reverse transcribed in a total volume of 25μl for 60 min at 37°C with M-MLV Reverse Transcriptase (Promega, Madison, WI, USA), using random primers.

### Real-time PCR

To examine PON2 and MIB-1 (Ki-67) gene expression quantitatively, we performed Real-Time PCR analyses using the CFX96 Real-Time PCR Detection System (Bio-Rad Laboratories, Hercules, CA, USA). cDNA generated, as previously described, was used as the template. To avoid false-positive results caused by amplification of contaminating genomic DNA in the cDNA preparation, all primers were selected to flank an intron. PCR efficiency was tested for both primer pairs and found to be close to 1. The primers used were (forward) 5′-TCGTGTATGACCCGAACAATCC-3′ and (reverse) 5′-AACTGTAGTCACTGTAGGCTTCTC-3′ for PON2, (forward) 5′-GACATCCGTATCCAGCTTCC-3′and (reverse) 5′-CCGTACAGGCTCATCAATAAC-3′ for MIB-1, and (forward) 5′-TCCTTCCTGGGCA TGGAGT-3′ and (reverse) 5′-AGCACTGTGTTGGC GTACAG-3′ for β-actin.

Genes were run in duplicate for 40 cycles at 95°C for 30 seconds and 58°C for 30 seconds, using SsoFast EvaGreen Supermix (Bio-Rad Laboratories, Hercules, CA, USA). All samples were tested in triplicate with the reference gene β-actin for data normalization. Direct detection of PCR products was monitored by measuring the fluorescence produced by EvaGreen dye binding to double strand DNA after every cycle. These measurements were then plotted against cycle numbers. The parameter threshold cycle (Ct) was defined as the cycle number at which the first detectable increase above the threshold in fluorescence was observed. The expression level of PON2 in both tissue and urine samples was expressed as ΔCt value, where ΔCt = Ct (PON2)- Ct (β-actin). A small ΔCt represents a high PON2 expression level, while a large ΔCt value is attributable to a low expression level. Fold changes in relative gene expression were calculated by 2^−ΔΔCt^ where ΔΔCt = mean-ΔCt (tumor tissues or urines from patients with BC) – mean-ΔCt (normal looking tissues or urines from healthy subjects).

Upon transfection, PON2 or MIB-1 expression levels in T24 cell line was evaluated by 2^−ΔΔCt^, where ΔCt = Ct (PON2 or MIB-1)- Ct (β-actin), and ΔΔCt = ΔCt (pcDNA3 or pcDNA3-PON2) - ΔCt (mock).

### Cell cultures

The human bladder cancer cell line T24, purchased from the America Type Culture Collection (ATCC, Rockville, MD, USA), was cultured in DMEM/F12 medium, supplemented with 10% fetal bovine serum and 50 μg/ml of gentamicin, at 37°C in a humidified 5% CO_2_ incubator.

### Cloning

Total RNA, isolated from T24 cells (5×10^5^) using the RNeasy Micro Kit (Qiagen, Hilden, Germany), was reverse transcribed with M-MLV Reverse Transcriptase (Promega, Madison, WI, USA) using random primers. 1 μl of the reaction mixture was then subjected to PCR with KOD Hot Start DNA Polymerase (Novagen, Darmstadt, Germany) in a total volume 50 μl, using the primers 5′-TCCGGATCCATGGGGCGGCTGGT-3′ (forward) and 5′-TTACTCGAGTTAGAGTTCACAAT-3′ (reverse) to amplify the human PON2 open reading frame (ORF) and to insert BamHI and XhoI restriction sites. The amplified and digested PCR product was cloned into the pcDNA3 plasmid vector (Life Technologies, Carlsbad, CA, USA) to obtain the plasmid construct pcDNA3-PON2.

### Transfection

T24 cells were seeded in 6-well plates (2.4×10^5^ cells/well) the day before transfection and were transfected with the pcDNA3-PON2 plasmid vector (3 μg per well). Control cells were transfected with the empty vector (pcDNA3) or treated with transfection reagent only (mock). Transfection was performed using FuGENE HD Transfection Reagent (Promega, Madison, WI, USA), according to the manufacturer's instructions. Forty-eight hours after transfection, cells were harvested and subjected to further analyses. The efficiency of PON2 overexpression in T24 cells was detected by Real-Time PCR, Western blot analysis and catalytic activity assay.

### Western blot analysis

Protein extracts from bladder tissues and T24 cells were prepared with lysis buffer (50 mM HEPES, pH 7.9, containing 150 mM NaCl, 0.5% Triton X-100, 1 mM phenylmethylsulfonyl fluoride and 2 μg/ml aprotinin). Aliquots of frozen tissue (20–40 mg) were suspended in 33 volumes of lysis buffer and homogenized on ice using Ultra-Turrax homogenizer (IKA, Staufen, Germany) at medium speed. Tissue homogenates were then centrifuged at 13000xg for 10 min at 4°C and the supernatant represented the total protein extract. Cell pellets (1×10^6^ cells) were suspended in 100 μl lysis buffer and homogenized by passing 3-5 times through a 30 gauche needle attached to a 1 ml syringe. After centrifugation at 13000xg for 10 min at 4°C, the supernatant containing the protein extract was collected. Samples containing 50 μg protein were subjected to 12.5% sodium dodecyl sulfate-polyacrylamide gel electrophoresis and transferred to polyvinylidene fluoride membranes. After regular blocking and washing, the membranes were incubated with rabbit polyclonal antibody against human PON2 (Sigma-Aldrich, St. Louis, MO, USA) (1:500 dilution), or with rabbit polyclonal antibody against human β-actin (Sigma-Aldrich, St. Louis, MO, USA) (1:1000 dilution), overnight at 4°C, followed by incubation (1:150000 dilution) with horseradish peroxidase (HRP)-conjugated goat anti-rabbit IgG (Sigma-Aldrich, St. Louis, MO, USA) for 1 h. PON2 protein was visualized using enhanced SuperSignal West Femto Maximum Sensitivity Substrate (Thermo Fisher Scientific, Waltham, MA, USA). The chemiluminescent signal of PON2 protein detected in blots was acquired using ChemiDoc XRS+ System (Bio-Rad Laboratories, Hercules, CA, USA).

### Protein assay

Protein concentration was measured by the Bradford method, using bovine serum albumin as the standard [21].

### PON2 lactonase activity

PON2 lactonase activity was measured using dihydrocoumarin (DHC) as substrate. Briefly, cell pellets (3×10^6^) were suspended in 200 μl of lysis buffer (25 mM Tris-HCl, pH 7.6, 1 mM CaCl_2_, 1mM PMSF, 1mM DTT, 2μg/ml aprotinin and 1% NP-40) and 0.5 volume glass beads. The suspension was vortexed for 2 min and then chilled on ice for 2 min. The homogenate was centrifuged at 16000xg for 10 min at 4°C, and the supernatant was kept on ice until assayed. The standard assay mixture contained 25 mM Tris-HCl, pH 7.6, 1 mM CaCl_2_, 5 mM DHC and 300 μg protein to reach a final volume of 1 ml. One unit of lactonase activity is equal to 1 μmol of DHC hydrolyzed/ml/min [22]. Experiments were repeated three times. Results were presented as mean values ± standard deviation of three independent experiments performed in triplicate.

### MTT assay

Cell proliferation was determined using a colori-metric assay with 3-(4,5-dimethylthiazol-2-yl)-2,5-diphenyl tetrazolium bromide (MTT). The MTT assay measures the conversion of MTT to insoluble formazan by dehydrogenase enzymes of the intact mitochondria of living cells. After transfection, T24 cells were seeded in 96-well plates (5×10^3^ cells/well). Cells were allowed to attach overnight and cell proliferation was evaluated (0, 24, 48 and 72 hours) by measuring the conversion of the tetrazolium salt MTT to formazan crystals. Briefly, 10 μl of MTT reagent (5 mg/ml in phosphate buffered saline) was added to the cells and incubated for 4 h at 37°C. The medium was removed and 200μl of isopropanol were added. The amount of formazan crystals formed correlates directly with the number of viable cells. The reaction product was quantified by measuring the absorbance at 570 nm using an ELISA plate reader. Experiments were repeated three times. Results were expressed as percentage of the control and presented as mean values ± standard deviation of three independent experiments performed in triplicate.

### Detection of intracellular oxidative stress

Intracellular oxidative stress was assayed through the oxidation of 2′,7′-dichlorodihydrofluorescein diacetate (DCFH_2_-DA) (Sigma-Aldrich, St. Louis, MO, USA). DCFH_2_-DA is readily taken up by cells and is subsequently de-esterified to 2′,7′- dichlorodihydrofluorescein (DCFH), which can be oxidized to dichlorofluorescein (DCF) by hydrogen peroxide, peroxynitrite, and other ROS or reactive nitrogen species. Cells were seeded on 96-well black plates with clear bottom (15×10^3^ cells per well) and allowed to adhere overnight. The medium was then removed and cells were pre-treated for 45 min at 37°C with DCFH_2_-DA (50μM) in the dark. The probe was added from a stock solution prepared in DMSO, which was added to the blank. Cells were washed to remove extracellular DCFH_2_-DA and then treated for 3 hours in the absence or presence of tert-butyl hydroperoxide (TBHP) (50, 100 and 200 μM). The fluorescence was measured on a fluorescence plate reader at Ex/Em= 485/535 nm [23, 24]. Experiments were repeated three times. Results were presented as mean values ± standard deviation of three independent experiments performed in triplicate.

### Statistical analysis

Data were analysed using IBM SPSS Statistics version 19 (IBM Corporation, New York, NY, USA). Significant differences between groups and correlation between variables were determined using the Wilcoxon, Kruskal-Wallis and the Mann-Whitney U tests. A p value <0.05 was considered as statistically significant.

## SUPPLEMENTARY MATERIALS TABLE



## Abbreviations

AdPON2: adenovirus-mediated expression of PON2
BC: bladder cancer
DCFH_2_-DA: 2′,7′-dichlorodihydrofluorescein diacetate
ER: endoplasmatic reticulum
MTT: 3-(4,5-dimethylthiazol-2-yl)-2,5-diphenyl tetrazolium bromide
PON: paraoxonase
PON2: paraoxonase-2
ROS: reactive oxygen species
TBHP: tert-butyl-hydroperoxide.
