# Supplementary material for: Exploring the role of Paraoxonase-2 in bladder cancer: analyses performed on tissue samples, urines and cell culturess
Source: Oncotarget. 2017 Feb 24;8(17):28785–95. doi: 10.18632/oncotarget.15674 (PMC5438691; doi:10.18632/oncotarget.15674)
Supplement: Supplementary file 1 [file oncotarget-08-28785-s001.pdf]

Exploring the role of Paraoxonase-2 in bladder cancer: analyses performed on tissue samples, urines and cell cultures

SUPPLEMENTARY DATA

Supplementary Data 1: Linkage between tissue PON2 expression and clinicopathologic parameters of BC patients

Parameter	Category	PON2 level [a]	p value [b]
Gender	Male	1.00 ± 0.19	0.294
	Female	2.03 ± 0.10	
Age (years)	<69	1.00 ± 0.19	0.161
	≥69	2.00 ± 0.32	
T classification	pT1-2	1.00 ± 0.19	0.328
	pT3-4	1.72 ± 0.31	
Lymph nodes	N0	1.00 ± 0.21	0.536
	N+	1.27 ± 0.20	

[a] Values represent mean ± standard deviation.
[b] Mann-Whitney U test was used for comparison of two groups.
